# Epidemiology and clinical aspect of mushroom poisonings in South Sardinia: A 10‐year retrospective analysis (2011–2021)

**DOI:** 10.1002/fsn3.3793

**Published:** 2023-10-25

**Authors:** Laura La Rosa, Serafina Corrias, Iginio Pintor, Sofia Cosentino

**Affiliations:** ^1^ Department of Medical Sciences and Public Health University of Cagliari Cagliari Italy; ^2^ Department of Prevention Food Hygiene and Nutrition Service, ASL Cagliari Cagliari Italy

**Keywords:** epidemiology, intoxications, mushroom poisoning, Sardinia

## Abstract

Mushroom poisoning is a severe problem of public health, however, information about its epidemiology and management is still limited. This is the first study focused on Sardinia that investigates data about mushroom poisoning describing epidemiology, clinical presentation, seasonality, and the most common involved species. In this retrospective study, we analyzed data recovered from the database of Mycological Inspectorates during a 10‐year period (2011–2021). Overall, 164 cases of mushroom poisoning have been identified, with significant peaks in autumn. The highest number of episodes of intoxication were recorded in Cagliari (64), followed by Carbonia (55) and Sanluri (45), although the annual distribution of cases among the provinces varied considerably in the study period. Thanks to the expertise of the Mycological Inspectorate service, the implicated species have been identified in 162 cases (98.78%); 81 cases were caused by toxic species, 60 by edible, and 45 by not edible species. *Omphalotus olearius* and *Agaricus xanthodermus* were the most represented toxic species (22% and 18%, respectively); *Boletus aereus* (18%) was the most frequent edible species, while Boletaceae were the prevalent not edible mushrooms. The data collected in South Sardinia over a period of 10‐years demonstrate how a correct and rapid recognition of mushroom poisoning is important to improve the prognosis of patients, however, there are still problems of lack of knowledge, on the part of the population, on the existence of the consultancy services. Because most illnesses from poisonous mushroom ingestion are preventable, increased public awareness about the potential dangers of mushroom poisoning is mandatory.

## Graphical abstract

This is the first study focused on South Sardinia that retrospectively investigates data about mushroom poisonings describing epidemiology, clinical presentation, seasonality, and most commonly involved species. The implicated species have been identified in 162 cases (98.78%); 81 cases were caused by toxic species, 60 by edible, and 45 by not edible species. Because most illnesses from poisonous mushroom ingestion are preventable, increased public awareness about the potential dangers of mushroom poisoning is mandatory.

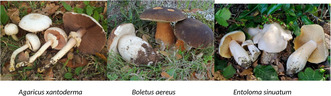

## 1 INTRODUCTION

In the field of food poisoning, those caused by the ingestion of mushrooms play a relevant epidemiological role, still representing a significant problem for public health in many countries (Somrithipol et al., 2022). Mushrooms have been part of the human diet for millennia (Wang et al., 2014), and their picking and consumption are common practice worldwide, especially in Asia, Europe, and the United States (Gawlikowski et al., 2015; Wu & Sun, 2004).

In Europe, there are around 2000 mushroom species, of which less than 100 are known to be poisonous (Cervellin et al., 2018; Eren et al., 2010). Poisonous species contain a variety of different toxins, whose ingestion causes mushroom poisoning (MP) or mycetism. It is relevant to note that most mushroom toxins are resistant to any processing method; consequently, the only way to avoid poisoning is to avoid the consumption of toxic species (Gawlikowski et al., 2015). Despite this, data in the literature indicate that poisoning by edible fungi occur much more often than intoxications by poisonous mushrooms (Govorushko et al., 2019), and this is due to the incorrect collection, transport, storage, and cooking of these edible species (Cervellin et al., 2018). However, the consequences of consumption of edible mushrooms are much less serious, usually limited to gastrointestinal disorders (Govorushko et al., 2019).

The yearly incidence of MP in the European country is not completely defined, since it is highly dependent on local ecology and gastronomic traditions, and it is believed to be underdiagnosed and underreported (Cervellin et al., 2018; Verma et al., 2014). Peintner et al. (2013) made a distinction between countries with strong traditions in consuming mushrooms (e.g., Italy and Slavic countries) and therefore called “mycophilic,” and countries (e.g., United Kingdom) named “mycophobic” where mushrooms are rarely picked and consumed. Obviously, mushroom poisonings are much more common in mycophilic countries, and this has led their governments to introduce appropriate legislative and control instruments concerning wild mushrooms (Govorushko et al., 2019).

In Italy, there are specific structures, the Mycological Inspectorates, established within the Food and Nutrition Services of the local healthcare administration, where expert mycologists offer their advice and consultancy services to citizens for the recognition of mushroom species, which is crucial since poisonings often occur after erroneous identification by amateur hunters (Cassidy et al., 2011). They also offer advice to healthcare professionals in hospitals in case of poisoning for the correct identification of the species involved.

The identification process requires specialist expertise, and the species is not easy to establish, since mushroom samples are not always available, and often there is a mixed meal assumption (more than one species consumed in the same meal) (Cervellin et al., 2018). Anyway, fast identification from a professional mycologist is invaluable to identify or exclude mortal species, and it influences clinical decisions regarding the need for hospitalization and specific treatments, also preventing unnecessary health costs (Cassidy et al., 2011; Eisenga et al., 1998).

Depending on the species consumed, there can be various clinical signs and symptoms ranging from gastrointestinal disturbances to organ failure and death (Keller et al., 2018; Lin & Wang, 2004; Schenk‐Jaeger et al., 2012). Patients with early symptoms (typically between 30 min and 6 h) normally have a favorable outcome, while delayed symptoms (after 6 h) are associated with a higher risk of serious complications (Diaz, 2005; Keller et al., 2018; Schmutz et al., 2018; Trueb et al., 2013). However, the consumption in the same meal of different species with both early and delayed toxicity may represent an additional source of confusion (Cervellin et al., 2018).

In the literature, several clinical case reports on MP have been published, while fewer studies have focused on characterizing the epidemiology of exposures (Gawlikowski et al., 2015; Kintziger et al., 2011). In Italy, data have been reported from Parma (Cervellin et al., 2018), the Poison Control Center of Milan (Assisi et al., 2019), Palermo (Venturella et al., 1994), Foggia (Pennisi et al., 2020) and Toscana (Centro di Riferimento Regionale sulle tossinfezioni alimentari, 2016).

The only study from Sardinia, specifically the province of Cagliari, has been from Sitta et al. (2020), who analyzed exclusively data related to mushroom species that caused intoxications in various Italian regions for a period of over 10 years.

Environmental and ecological characteristics in Sardinia make this region particularly rich in mushroom species, and over the years there has been a strong increase in mushroom gatherers (Brotzu, 2007).

Since there are no previous studies on this topic in Sardinia, the aim of this report is to analyze retrospectively data about mushroom poisonings in the three South Sardinia provinces (Cagliari, Carbonia, Sanluri) between 2011 and 2021, describing epidemiology, clinical presentation of patients, seasonality, most common involved species and principal mistakes in their consumption, in order to get an overview of the situation and be able to implement proper prevention measures.

## 2 MATERIALS AND METHODS

In the period between 1998 and 2010, the local healthcare administration in Sardinia instituted a mycological consultation service (Mycological Inspectorate, MI) in each of the seven provinces: Cagliari, Carbonia, Sanluri, Lanusei, Olbia, Oristano, and Sassari.

Mycologists working in these structures, in addition to other functions, offer mycological advice to hospitals and emergency departments that need support in case of mushroom poisoning (MP).

In this retrospective study, we analyzed data related to mushroom intoxications during a 10‐year period in the provinces of Cagliari, Carbonia, and Sanluri, located in the Southern part of Sardinia.

Data was recovered from the database of MI of each province, and we included all records from January 1, 2011 to December 31, 2021. The following parameters (if available) were extracted: date, province, age of the patient, sex, mushroom species and how they were obtained (e.g., self‐harvest, given by others, purchased), cooking method, place of consumption, symptoms, latent period from mushroom ingestion to onset of symptoms, syndrome, hospitalization length, and outcome. In case of poisoning caused by edible mushroom, the circumstance of collection, storage, and consume was often reported.

We calculated the annual and monthly distribution of MP, number of cases by province and year, number and percentage of poisoning caused by different edible and toxic species as well as the percentage of poisoning due to the collection method and the circumstance of collection and consume. We described the clinical presentation of the patients also by mushroom types and analyzed data on gender and age groups.

Mushroom biology is particularly influenced by environmental parameters like temperature and rainfall, thus climate characteristics influence the seasonality of MP episodes. Due to its geographic position, Sardinia has a typically Mediterranean climate, with dry summers and relatively mild and rainy winters (Canu et al., 2015). The peak temperature in summer is higher than 35°C, the average annual rainfall is less than 500 mm in the coast and 1300 mm in the mountains (Cardil et al., 2014), and occurs mostly in fall with peak in October and November.

## 3 RESULTS AND DISCUSSION

Mushroom poisoning (MP) is an important public health issue in many countries, although its incidence is variable, being higher in those where wild mushrooms traditionally play an important role in food, such as Japan, Europe, and China (Chen et al., 2021; Govorushko et al., 2019).

In our retrospective covering a 10‐year period (2011–2021), 164 cases of MP have been identified in South Sardinia. In a recent 21‐years retrospective analysis, Cervellin et al. (2018) reported 443 cases of MP in the province of Parma, Northern Italy.

**FIGURE 1:**
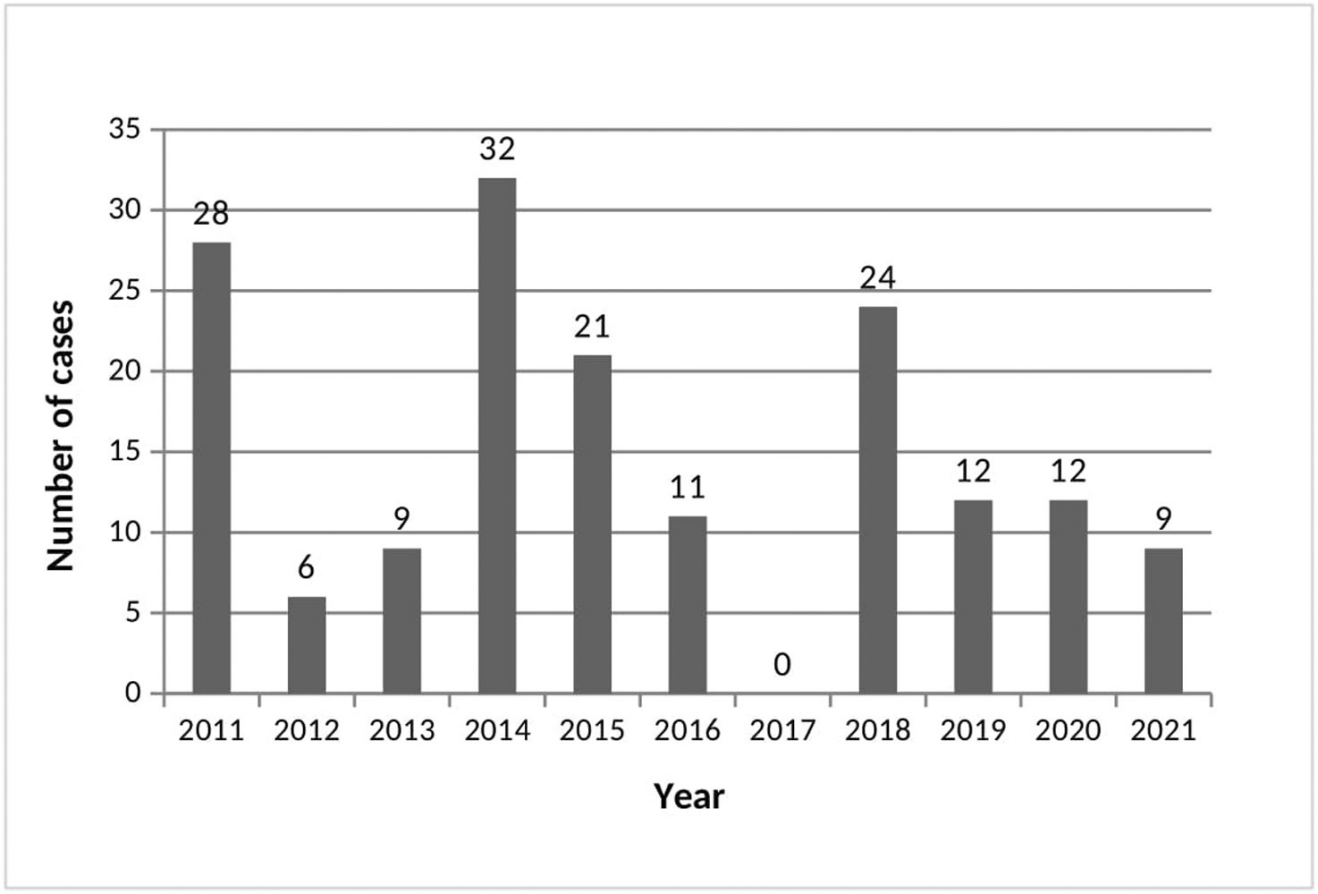
Annual distribution of mushroom poisoning in South Sardinia.

Figure 1 shows the distribution of MP in South Sardinia by year. The annual number of episodes during the study period varied considerably, ranging from 0 in 2017 to a peak of 32 in 2014. This difference is probably attributable to the different rainfall, in fact in 2017 the precipitation levels were significantly lower compared to the other years (data not shown). Similar fluctuations generally attributable to climatic conditions have been highlighted by other authors (Cervellin et al., 2018; Keller et al., 2018; Somrithipol et al., 2022).

**FIGURE 2:**
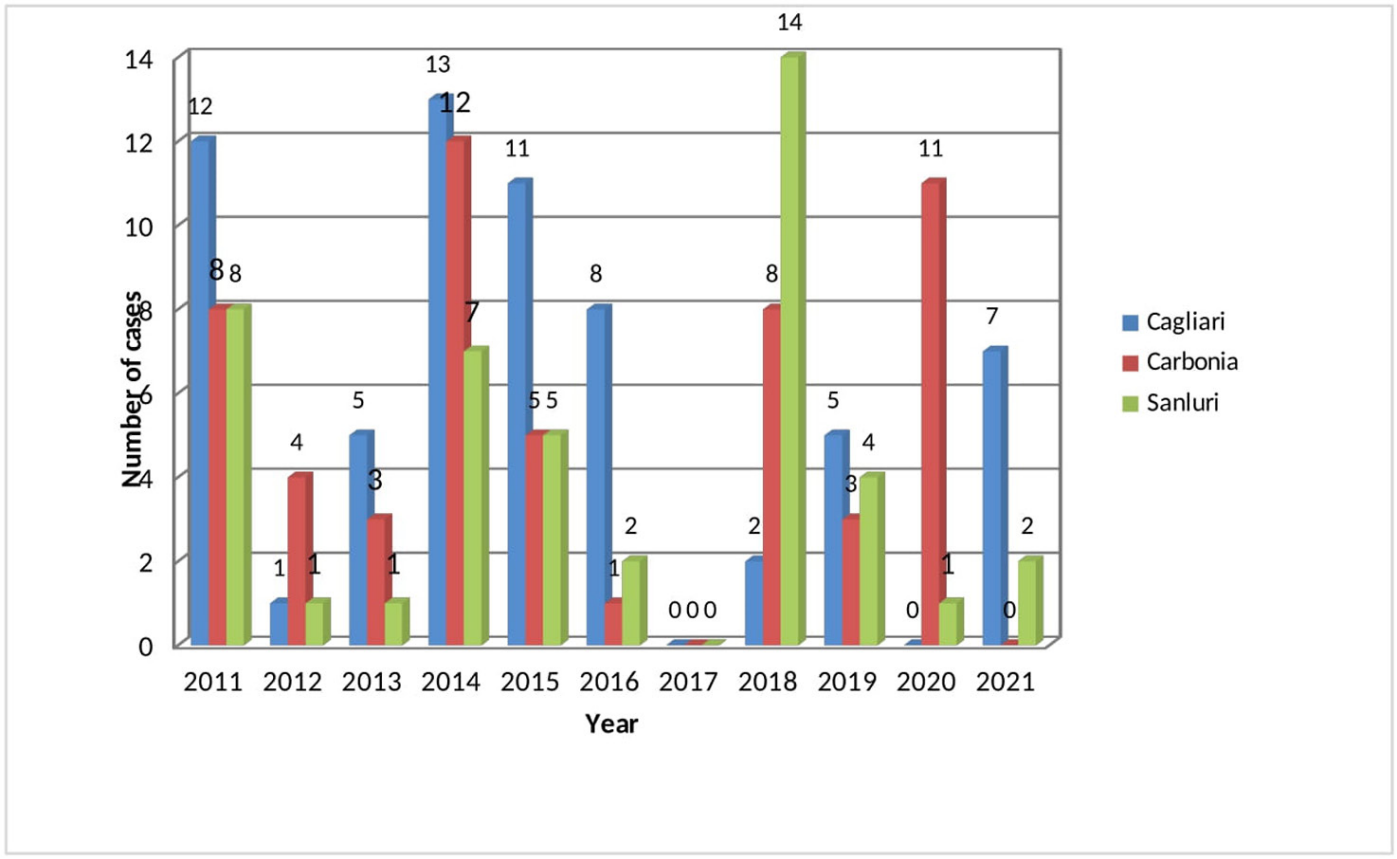
Annual distribution of mushroom poisoning by province and year.

Figure 2 reports the distribution of MP by year and province. The highest number of episodes of intoxication were recorded in Cagliari (64), followed by Carbonia (55) and Sanluri (45), although the annual distribution of cases among the provinces varied considerably in the study period. In the only previous study analyzing, to our knowledge, data on mushroom species that cause poisoning in Sardinia, 46 cases were reported for the area of Cagliari in the years 2008–2017 (Sitta et al., 2020).

**FIGURE 3:**
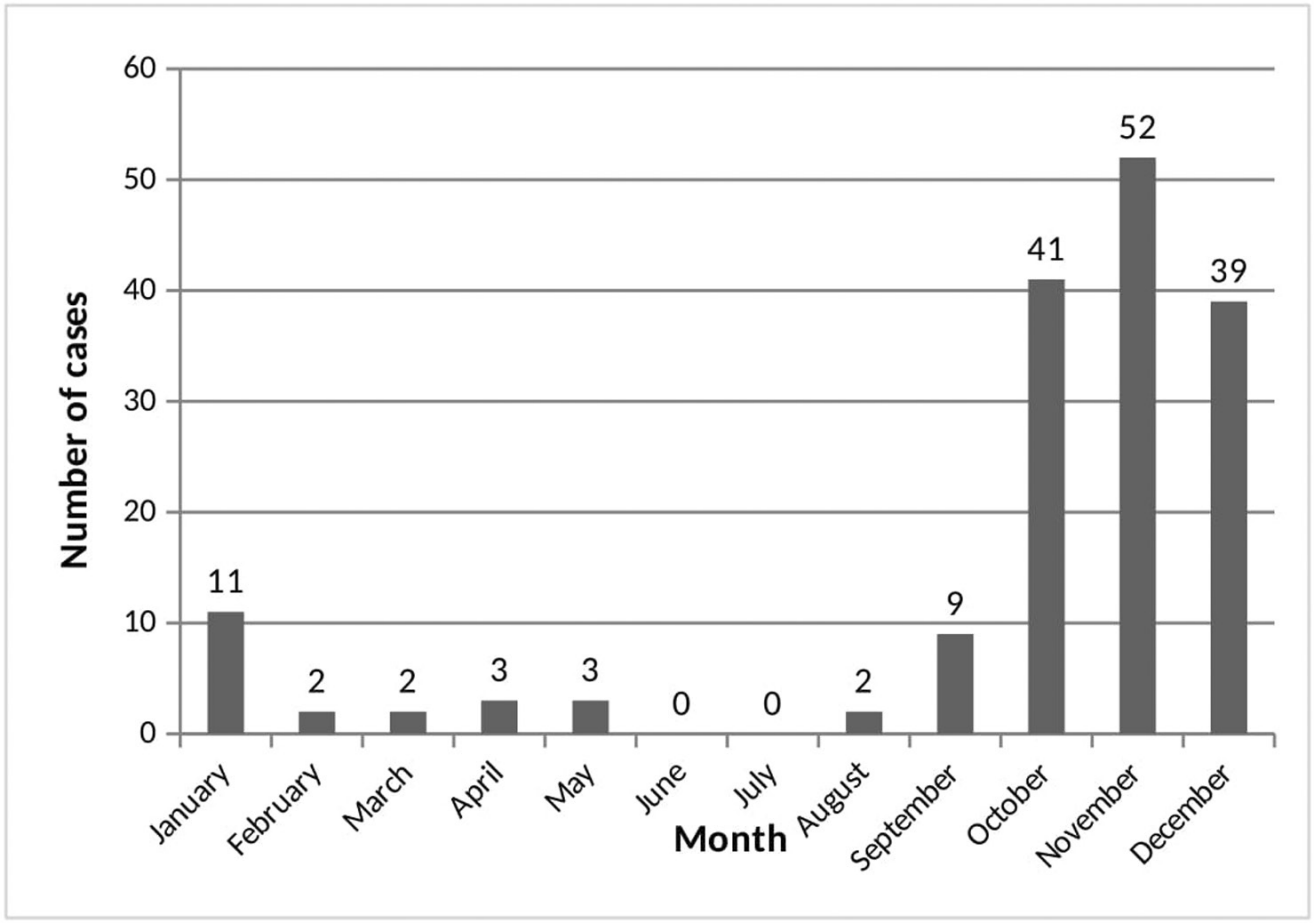
Monthly distribution of mushroom poisoning. Bars represent the sum of episodes presenting during the same month throughout the study period (2011–2021).

Despite the yearly differences, MP occurred each year most frequently in autumn, with a total of 141 cases (86%) distributed between September and December (Figure 3), probably attributable to the climate conditions typical of this period. A seasonality in the occurrence of MP has been reported by many studies, generally showing peaks in autumn related to rainfall abundance (Cervellin et al., 2018; Keller et al., 2018), although an additional small spring peak has been observed by some authors (Gawlikowski et al., 2015; Schmutz et al., 2018). In the study by Lewinsohn et al. (2023), MP significantly occurred in rainy winters.

As indicated in Table 1, in the studied population all ages were represented, ranging from 5 to 90 years, and the age group more represented was 18–64 years with 116 cases. In 6 cases, the patients were children and adolescents who tended to be unintentionally poisoned by eating wild mushrooms picked from adults, despite the Italian Health Ministry indication to avoid mushroom eating for children under 12 years old. Even though it is widely acknowledged that mushrooms should be avoided in children due to their potential toxicity, episodes of children intoxication have been reported in Italy as well as other countries, often related to spontaneous consumption of mushrooms found in the fields surrounding schools or houses (Cervellin et al., 2018; Cevik & Unluoglu, 2014).

**TABLE 1:** Characteristics of reported mushroom poisoning patients by age group.

Characteristic	Age groups in years				ND (*n* = 3) *n* (%)
	0–6 (*n* = 1) *n* (%)	7–17 (*n* = 5) *n* (%)	18–64 (*n* = 116) *n* (%)	≥65 (*n* = 39) *n* (%)	
*Gender*					
Male	0	3 (60)	63 (54.31)	26 (66.66)	0
Female	1 (100)	2 (40)	53 (45.69)	13 (33.34)	3 (100)
*Source of mushrooms*					
Self‐harvest	1 (100)	5 (100)	95 (81.89)	33 (84.61)	2 (66.67)
Purchased in shop	0	0	2 (1.74)	0	0
Purchased from a person	0	0	19 (16.37)	6 (15.39)	1 (33.33)
*Cooking method*					
Boiled	1 (100)	3 (60)	58 (49.15)	21 (52.50)	1 (33.33)
Fried	0	0	13 (11.03)	9 (22.50)	1 (33.33)
Roasted	0	0	33 (27.96)	6 (15.00)	1 (33.33)
Raw	0	2 (40)	14 (11.86)	2 (5)	0
ND	0	0	0	2 (5)	0
*Latent period*					
<6 h	1 (100)	5 (100)	73 (62.94)	23 (58.97)	1 (33.33)
>6 h	0	0	11 (9.48)	5 (12.82)	1 (33.33)
ND	0	0	32 (27.58)	11 (28.21)	1 (33.33)

Abbreviation: ND, Not determined.

Overall, 92 (56.1%) intoxicated were males, and 72 (43.9%) females. Epidemiological inequality in gender has been reported by several studies from Thailand (Somrithipol et al., 2022), Anatolia (Cevik & Unluoglu, 2014), and Switzerland (Keller et al., 2018) where MP was more frequent in females, whereas others showed a higher number of male patients, in agreement with our findings (Jiang et al., 2018; Kintziger et al., 2011; Lewinsohn et al., 2023).

Regarding the source of mushrooms, in 136 cases (83%) the mushrooms were self‐harvested, in 26 cases (15.8%) purchased by a person, and only in 2 cases (1.2%) purchased in a shop, in agreement with the data from similar studies where about three quarters of MP events were related to adults collecting and eating wild mushroom intentionally (Cevik & Unluoglu, 2014; Keller et al., 2018). The high percentage of MP deriving from self‐harvesting suggests a possible lack of knowledge on the citizen's part about the existence, on a large part of Italian territory, of the Mycological Inspectorates that offer consultancy services for the correct identification of mushrooms.

In our study, many cooking methods were used for the mushroom before ingestion, boiling, and roasting being the most common (82 and 39 cases, respectively). In 18 cases (11%) mushrooms were consumed raw.

In MP an important factor is represented by the interval between the ingestion and the occurrence of clinical manifestations. Similar to other studies (Gawlikowski et al., 2015; Keller et al., 2018; Schmutz et al., 2018) most cases (62.8%) presented with early symptoms (within the first 6 h of ingestion) which are generally associated with a favorable outcome, 17 cases (10.4%) had delayed symptoms (after >6 h), and in 44 (26.8%) cases data were not available.

The adverse effects of MP range from mild gastrointestinal symptoms to organ failure and death. In our study, most cases (79.3%) were characterized by gastrointestinal disturbances, and the remaining 20.7% included neurological disorders (Tables 2 and 3) but no death or severe complications were observed. The most common symptoms were vomiting, nausea, diarrhea and cramps, and most hospitalized subjects were discharged within 1–2 days of admission, in line with the literature data (Eren et al., 2010; Gawlikowski et al., 2015; Keller et al., 2018). Most severe cases were those with delayed symptoms.

**TABLE 2:** Clinical characteristics of mushroom poisoning.

Symptoms	*n* (%)
*Gastrointestinal*	130 (79.3%)
Abdominal cramps	47 (28.6%)
Colic	9 (5.5%)
Diarrhea	68 (41.5%)
Nausea	77 (47%)
Stomach ache	4 (2.4%)
Vomiting	117 (71.3%)
*Nerurological*	34 (20.75)
Agitation	3 (1.8%)
Dyspnea	1 (0.6%)
Fever	4 (2.4%)
Head ache	1 (0.6%)
Muscular cramps	3 (1.8%)
Sickness	2 (1.2%)
Sweat	14 (8.5%)
Tremor	10 (6%)
Vertigo	8 (4.9%)

**TABLE 3:** Suspected mushroom types, clinical presentation and edibility.

Mushroom type	Edibility	Cases	Delay of symptoms (h) (mean)	Period of hospitalization (days) (mean)	Symptoms
*Omphalotus olearius*	*Toxic*	18	3.5	2	Colic, cramps, diarrhea, nausea, sweat, tremor, vomiting
*Agaricus xanthodermus*	*Toxic*	15	2	1	Abdominal pain, abdominal cramps, dyspnea, diarrhea, nausea, tremor, vomiting, vertigo
*Entoloma sinuatum*	*Toxic*	14	1	1	Diarrhea, head ache, nausea, stomachache, sweat, tremor, vertigo, vomiting
*Boletus aereus*	*Edible*	11	3	1	Abdominal pain, abdominal cramps, diarrhea, nausea, tremor, vomiting, vertigo
*Chlorophyllum rachodes*	*Not Edible*	9	1	1	Abdominal cramps, colic, diarrhea, nausea, vomiting
*Boletus* sp.	*Edible*	8	4	ND	Cramps, diarrhea, nausea, stomachache, tremor, vomiting
*Gymnopus fusipes*	*Toxic*	7	1	2	Nausea, vomiting
*Leccinellum lepidum*	*Edible*	7	6	1	Abdominal cramps, diarrhea, nausea, tremor, vomiting
*Rubroboletus pulchrotinctus*	*Toxic*	6	6	3	Cramps, diarrhea, fever, nausea, sweat, stomachache, tremor, vertigo, vomiting
*Armillaria mellea*	*Edible*	6	1.5	1	Abdominal cramps, colic, diarrhea, fever, nausea, vomiting
*Agaricus* sp.	*Not Edible*	6	13	ND	Abdominal pain, diarrhea, nausea, vomiting
*Clitocybe dealbata*	*Toxic*	5	ND	1	ND
*Leccinellum corsicum*	*Edible*	4	2.5	1	Diarrhea, nausea, vomiting
*Clitocybe nebularis*	*Toxic*	4	2.5	2	Diarrhea, nausea, vomiting
*Boletus edulis*	*Edible*	4	3.5	1	Abdominal cramps, nausea, vomiting
*Boletus aestivalis*	*Edible*	4	2	1	Cramps, diarrhea, nausea, vomiting
*Amanita crocea*	*Edible*	3	2	1	Agitation, nausea, vomiting
*Amanita gilbertii*	*Not Edible*	3	10	12	Abdominal pain, muscolar cramps, diarrhea, nausea, sweat, tremor, vomiting
*Amanita phalloides*	*Toxic*	3	ND	ND	Vomiting
*Hemileccinum impolitum*	*Edible*	3	6.5	1	Abdominal pain, diarrhea, nausea, vomiting
*Caloboletus radicans*	*Toxic*	3	4	1	Cramps, diarrhea, fever, vertigo, vomiting
*Lepista nuda*	*Edible*	3	ND	ND	Cramps, nausea, vomiting
*Lepiota brunneoincarnata*	*Toxic*	3	13	ND	Diarrhea, vomiting
*Pleurotus* sp.	*Edible*	3	ND	ND	Cramps, vomiting
*Pleurotus eryngii*	*Edible*	3	2.5	1	Abdominal cramps, diarrhea, nausea, vertigo, vomiting
*Rubroboletus lupinus*	*Toxic*	2	1.5	2	Nausea, vomiting
*Macrolepiota procera*	*Edible*	2	1	2	Sickness
*Leccinellum corsicum*	*Edible*	2	2	ND	Cramps, diarrhea, nausea, vomiting
*Lactarius acerrimus*	*Toxic*	2	2	1	Cramps, diarrhea, nausea, vomiting
*Hygrophorus russula*	*Not Edible*	2	1	1	Diarrhea, nausea, vomiting
*Infundibulicybe geotropa*	*Edible*	2	2	1	Abdominal pain, diarrhea, nausea, vomiting
*Cantharellus cibarius*	*Edible*	2	16	1	Abdominal pain, diarrhea, nausea, vomiting
*Armillaria* sp.	*Not Edible*	2	ND	ND	Abdominal pain, diarrhea, nausea, vomiting
*Agaricus bernardii*	*Not Edible*	1	12	1	Abdominal pain, diarrhea, nausea, vomiting
*Agaricus bitorquis*	*Edible*	1	2	2	Cramps, nausea
*Agaricus bresadolanus*	*Toxic*	1	2	1	Nausea, vomiting
*Agaricus campestris*	*Edible*	1	12	1	Cramps, colic
*Agaricus moelleri*	*Toxic*	1	5	ND	Cramps, diarrhea, vomiting
*Amanita caesarea*	*Edible*	1	11	ND	Fever, cramps, sweat, tremor
*Boletus sez. luridi*	*Not Edible*	1	6	1	Agitation, nausea, vomiting, vertigo, tremor
*Infundibulicybe gibba*	*Edible*	1	1	1	Sickness
*Clitocybe cerussata*	*Toxic*	1	1	1	Colic, diarrhea, nausea, vomiting
*Hygrophorus penarioides*	*Edible*	1	2	1	Diarrhea, nausea, vomiting, tremor
*Lentinus tigrinus*	*Not Edible*	1	2	1	Diarrhea, vomiting
*Macrolepiota* sp.	*Not Edible*	1	4	ND	Stomachache
*Melanoleuca malaleuca*	*Edible*	1	ND	1	ND
*Suillus granulatus*	*Edible*	1	2	1	Diarrhea, nausea, vomiting, colic
*Suillus* sp.	*Edible*	1	2	ND	Cramps
ND		2	3	ND	Cramps, diarrhea, sweat, vomiting

Abbreviation: ND, Not Determined.

**FIGURE 4:**
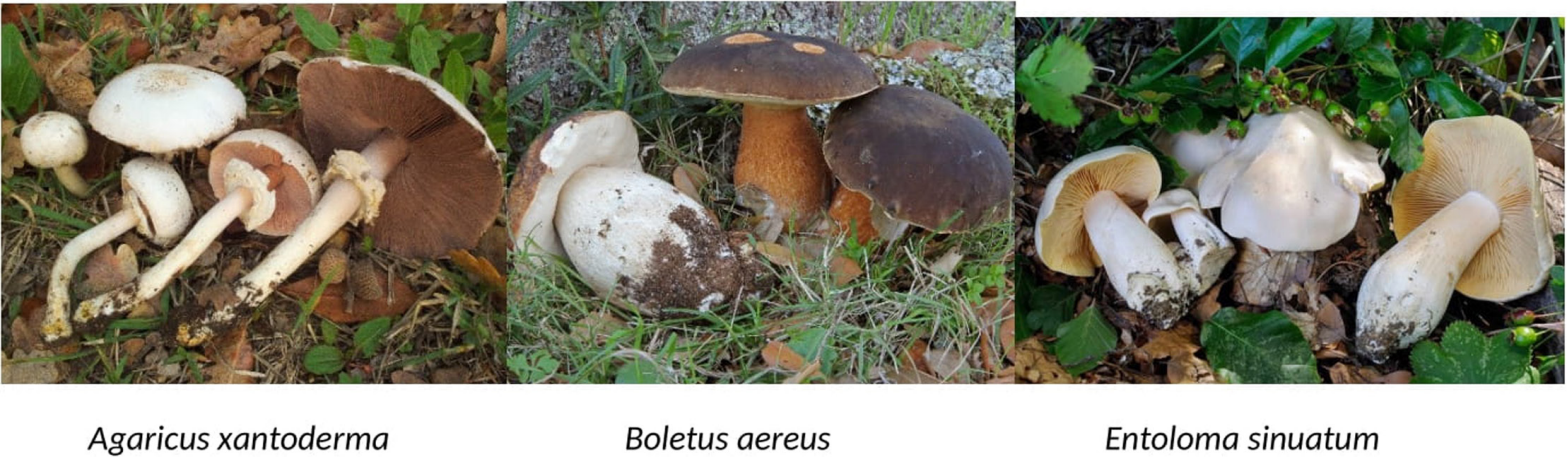
Three topmost poisonous mushroom species of our survey.

In MP, the correct identification of mushroom species is of crucial importance for the management of potentially life‐threatening cases but is often difficult, with identification rates generally low (10%–27%) in studies were obtaining a mycologist opinion was reported as difficult (Schmutz et al., 2018; Unluoglou & Tayfur, 2003). In our study, we were able to identify the mushroom species or family in almost all cases (98.78%), in agreement with the Italian study of Cervellin et al. (2018), presumably due to the rapidly available mycologist service in case of suspected MP. Table 3 shows mushroom types according to clinical presentation and edibility while Figure 4 shows the three topmost poisonous species. Overall, *Boletus* and *Agaricus* were the genera most frequently identified, the latter being highly consumed in the province of Cagliari; 81 cases were caused by toxic species, 60 by edible, and 45 by not edible species. *Omphalotus olearius* and *Agaricus xanthodermus* were the most represented toxic species (22% and 18% respectively); *Boletus aereus* (18%) was the most frequent edible species, while Boletaceae were the prevalent not edible mushrooms. A high incidence of MP caused by edible species ingestion has been reported by several studies and has been generally attributed to inappropriate transport, storage, and cooking of mushrooms (Cervellin et al., 2018; Gawlikowski et al., 2015; Keller et al., 2018).

## 4 CONCLUSION

Although mushroom poisoning is relatively uncommon, it currently remains a harmful and life‐threatening condition. In fact, the unsafe species are various, as well as the potential clinical presentations. The data collected in South Sardinia over a period of 10‐years demonstrate how a correct and rapid recognition of possible mushroom poisoning can improve the prognosis of patients. Despite the presence of Mycological Inspectorates on a large part of the national territory, however, there are still problems of lack of communication between hospital doctors and mycologists, as well as lack of knowledge, on the part of the population, on the existence of these consultancy services greatly helpful in the identification of the harmful species. Because most illnesses from poisonous mushroom ingestion are preventable, increased public awareness about the potential dangers of mushroom poisoning is mandatory. Some possible strategies to reach this goal may be (i) increase prevention activity through mycological exhibitions directed to the population and (ii) provide specific training to doctors and hospital staff for the management of mycological intoxications.

## AUTHOR CONTRIBUTIONS

**Laura La Rosa:** Data curation (lead); formal analysis (lead); investigation (equal); writing – original draft (lead). **Serafina Corrias:** Investigation (equal); methodology (lead). **Iginio Pintor:** Conceptualization (equal); supervision (equal); validation (lead). **Sofia Cosentino:** Conceptualization (equal); supervision (equal); visualization (lead); writing – review and editing (lead).

## CONFLICT OF INTEREST STATEMENT

The authors declare no conflict of interest.

## Data Availability

The data used to support the findings of this study are available from the corresponding author upon request.
